# Host niche partitioning and coexistence in *Amoebophrya* and *Parvilucifera* parasitoids infecting dinoflagellates

**DOI:** 10.1093/ismeco/ycaf126

**Published:** 2025-07-29

**Authors:** Bora Lee, Boo Seong Jeon, Myung Gil Park

**Affiliations:** Research Institute for Basic Science, Chonnam National University, Gwangju 61186, Jeollanam-do, Republic of Korea; Ecological Risk Research Department, Korea Institute of Ocean Science and Technology, Geoje 53201, Gyeongsangnam-do, Republic of Korea; Research Institute for Basic Science, Chonnam National University, Gwangju 61186, Jeollanam-do, Republic of Korea; Laboratory of Harmful Algal Blooms Ecophysiology, Department of Oceanography, Chonnam National University, Gwangju 61186, Jeollanam-do, Republic of Korea

**Keywords:** coexistence, competition, niche overlap, Syndiniales, Parviluciferaceae

## Abstract

Although much attention has been paid to defining the ecological niches of phytoplankton, those of marine parasites and/or parasitoids remain unclear. In this study, we aimed to define the ecological niches of *Amoebophrya* and *Parvilucifera* parasitoids infecting dinoflagellates. By performing high-frequency (i.e. daily) time series monitoring over 411 days at a temperate coastal site in Jinhae Bay located on the southern coast of the Korean Peninsula, we isolated infected dinoflagellates and performed the outlying mean index analysis, a multivariate technique that identifies realized niches in field data. Our findings revealed distinct niche properties: *Amoebophrya* spp. (i.e. Syndiniales clades II-C2, -C3, and -C4) exhibited high marginality, while *Parvilucifera* spp. exhibited moderate marginality. These findings suggest that the latter species occupy more typical environmental conditions. Despite their shared realized niches, the parasitoids exhibited varying levels of niche overlap and used host niche partitioning to reduce competition and coexist. These findings enhance our understanding of the distribution and dynamics of marine parasitoids in the field.

## Introduction

Interactions involving several parasite species (i.e. multi-parasite hosts, which harbor multiple parasites) or several host species (i.e. multi-host parasites, which infect multiple host species) are common in nature [1]. For example, in marine planktonic food webs, such interactions are observed in dinoflagellates. Dinoflagellates themselves can function as parasites. Approximately 150 dinoflagellate species belonging to 35 genera are parasites that utilize an array of organisms, such as protists, algae, invertebrates, and vertebrates, as hosts [2–5]. In turn, dinoflagellates could host various eukaryotic parasites, including parasitic dinoflagellates belonging to the genus *Amoebophrya* [6], perkinsozoan flagellates belonging to the families Parviluciferaceae and Pararosariidae [7], chytrids [8–11], and early-diverging oomycetes [12]. Therefore, these parasites (hereafter referred to as parasitoids because they complete their life cycle by ultimately killing the host) are expected to strongly compete for the same resource (i.e. dinoflagellate host) in the field, probably due to host niche overlap. However, this possibility has not been explored so far.

Although several attempts have been made to analyze the ecological niche of phytoplankton, including harmful algal bloom species [13–19], the ecological niche of eukaryotic parasitoids, particularly those infecting dinoflagellates, remains poorly understood. The Hutchinson’s niche is defined as an n-dimensional hypervolume that can distinguish between fundamental and realized niches (NRs) [20]. A fundamental niche is a favorable condition determined by all environmental variables. In addition, it has no biological interactions (pre-interactive, precompetitive, or virtual niche). An NR is a subset of a fundamental niche in which species can prevail even though biological interactions (e.g. predation, parasitism, or competition) are considered (post-interactive or post-competitive niche). Therefore, theoretically, an NR is always narrower than a fundamental niche [21]. To the best of our knowledge, only one previous study [22] has addressed the ecological niche of parasitic dinoflagellates belonging to the genus *Amoebophrya*. The authors of that study identified at least eight *Amoebophrya* ribotypes in the Penzé estuary, France. They found that most of these ribotypes coexisted during dinoflagellate blooms and shared the same ecological niche, indicating potential competition for the same resources (i.e. hosts). However, the authors did not assess how the coexisting *Amoebophrya* ribotypes compete for the same hosts or whether they exhibit host niche separation.

In the present study, we aimed to define the ecological niche of *Amoebophrya* and *Parvilucifera* parasitoids, which are known to use dinoflagellates as hosts. Both these parasitoids exhibit a similar life cycle consisting of an infective, free-living stage, a growth stage, and a proliferative stage [4, 23]. During the growth stage within the host cell, *Amoebophrya* spp. consume the host contents and form a multinucleate, multiflagellate trophont; this is referred to as the beehive stage. During the proliferative stage, the mature trophont exits the host cell in a vermiform shape, persists for a few hours, and undergoes cytokinesis to form dinospores. Unlike *Amoebophrya* spp., *Parvilucifera* spp. form a spherical trophocyte within the host cell during the growth stage. After consuming the host contents, they undergo karyokinesis and cytokinesis to form new zoospores within a sporangium. The newly generated zoospores are then released through apertures, which are covered by opercula. *Amoebophrya* spp. usually have a narrower host range than *Parvilucifera* spp. [24, 25]; therefore, their ecological niche is expected to be narrower than that of *Parvilucifera* spp. Furthermore, considering the close physical relationship between the hosts and parasitoids, we hypothesized that the ecological niches of the parasitoids would be similar or almost identical to those of their hosts. To test this hypothesis, we addressed the following specific questions in this study: do the parasitoids share the same ecological niches with their hosts? Do the parasitoids share the same or similar niches with each other? If yes, how do they compete for the use of the same resources (i.e. hosts) under such a niche overlap? To answer these questions, we isolated individual dinoflagellates infected by the parasitoids from field samples by performing high-frequency (i.e. daily) time series monitoring for 411 days between April 2020 and May 2021 at a temperate coastal site in Jinhae Bay located on the southern coast of the Korean Peninsula. Such high-frequency time series monitoring could provide deeper insights into the ecological niches of the parasitoids if they coexist in the same environment. This is because such a sampling strategy covers various environmental conditions that the parasitoids may encounter over the course of more than a year.

## Materials and methods

### Field sampling and quantification of dinoflagellates infected by parasitoids

Samples were collected from a fixed monitoring site [Laboratory of Harmful Algal Blooms Ecophysiology (LOHABE) time-series monitoring station; 35°07′40″N, 128°41′54″E; Supplementary Fig. S1] from April 2020 to May 2021. The following environmental variables were measured in this study: water temperature (°C), salinity, chlorophyll fluorescence (μg L^−1^), tidal coefficient, wind speed (m s^−1^), precipitation, stability ratio (Rρ), and concentrations of dissolved inorganic nutrients, (i.e. ammonium (NH_4_), nitrate (NO_3_), nitrite (NO_2_), silicate [Si(OH)_4_], and phosphate (PO_4_) (μmol L^−1^). The RBR concerto3 C.T.D. (conductivity, temperature, and depth profiler; RBR Ltd., Ottawa, Canada) with a fluorometer (Sea-Bird Scientific, Bellevue, WA, USA) was used to measure water temperature, salinity, depth, and chlorophyll fluorescence. Precipitation and wind speed data were obtained from the Korea Meteorological Administration (KMA). Rρ was computed using the method of Pond and Pickard (1983) [26] based on water temperature and salinity data. The following equation was used:


$$ \mathrm{R}\mathrm{\rho} =-1/\mathrm{\rho}\ \left(\delta \rho /\delta z\right) $$

Here, Rρ represents the stability parameter (m^−1^), ρ is the density (kg m^−3^) of water, and z is the depth (m). Rρ > 0 denotes a stable state, Rρ = 0 denotes a neutral state, and Rρ < 0 denotes an unstable state. For analyzing the concentrations of dissolved inorganic nutrients, ~300 ml of the water sample was collected in a polyethylene bottle and filtered through a GF/F filter paper (Whatman, UK). Aliquots of the filtrate were then stored at −20°C. The concentrations of dissolved inorganic nutrients, i.e. NO_3_, NO_2_, Si(OH)_4_, PO_4_, and NH_4_, were analyzed using an autoanalyzer (New QuAAtro39, SEAL Analytical, Southampton, UK). The accuracy of the concentrations was verified before the analysis of the samples by using the certified reference material (CRM) RMNS (KANSO, Japan), according to the method of Kim *et al.* (2022) [27].

To quantify the abundance of dinoflagellates, a surface seawater sample was collected using a bucket. In total, 500 ml of the sample was fixed with neutral Lugol’s iodine solution (final concentration, 2%) and allowed to sediment for 24 h. The fixed sample was then concentrated to 50 ml, and a 1 ml aliquot was loaded onto a Sedgewick−Rafter counting chamber using an automated pipette. All dinoflagellate cells were counted using an inverted microscope (AX10, Carl Zeiss Inc., Hallbergmoos, Germany). To quantify the number of dinoflagellates infected by the two parasitoids, 40 l of surface seawater was collected using a bucket and then filtered through 200-μm and 20-μm meshes to remove particles, large zooplankton, and free-swimming parasitic spores. The filtered sample was concentrated to 50 ml. To quantify the abundance of *Amoebophrya* spp., 20 ml of the concentrated sample was fixed with glutaraldehyde at a final concentration of 1% in a Petri dish (SPL Life Sciences, Korea). *Amoebophrya* spp. were detected within the host cells by their natural bright green autofluorescence [28], indicative of infection. They were counted using an epifluorescence microscope under blue light excitation (Filter Set 09, excitation BP 450–490, beam splitter FT 510, emission LP 515). To quantify *Parvilucifera* infection, 20 ml of the concentrated sample was placed in a Petri dish (50 × 15 mm) and incubated at 20°C for 20 h as per the method reported by Jeon and Park [29]. This allowed *Parvilucifera* spp. to grow sufficiently and exhibit signs of infection from the middle to late trophocytes stage to the early middle sporocyte stage. This also enabled the confirmation of the host species. The infected dinoflagellate hosts were photographed at 200× magnification using a mobile camera (Galaxy S21, Samsung Electronics, Suwon, Korea).

### Deoxyribonucleic acid extraction and polymerase chain reaction amplification

To isolate dinoflagellates infected by *Amoebophrya* spp., a plankton net with a pore size of 20 μm was vertically towed from the bottom to the surface daily. In total, 50-ml aliquots were then fixed with neutral Lugol’s iodine solution (final concentration, 2%). The fixed samples were poured into plant culture dishes (SPL Life Sciences, Korea) to allow the cells to settle. Host cells in the late stages of *Amoebophrya* infection were randomly isolated using drawn Pasteur pipettes and viewed using an inverted microscope or a stereoscope (Discovery. V8, Carl Zeiss Inc., Hallbergmoos, Germany). The isolated single cells were then washed several times with sterilized seawater, placed into a polymerase chain reaction (PCR) tube, and stored at −80°C for a day. The samples were thawed at room temperature, and deoxyribonucleic acid (DNA) was extracted using Chelex 100 resin (100–200 mesh, sodium form, Bio-Rad Laboratories, Hercules, CA, USA). Subsequently, 20 μl of 10% Chelex solution was added to the samples and incubated at 95°C for 1 h. The samples were then centrifuged at 8000 rpm at 4°C for 1 min. The supernatant was transferred to a new PCR tube and used as a template to amplify the nuclear small subunit (SSU) ribosomal DNA (rDNA). PCR amplification was performed using the forward primer ALV1 [30] and the reverse primer EUK329r [31]. The PCR mixture was amplified in a total volume of 25 μl using DiastarTM Taq DNA polymerase (SolGent Co., Daejeon, Korea). The PCR program was as follows: an initial cycle of 5 min at 95°C; 40 cycles of 20 s at 95°C, 20 s at 55°C, 1 min 20 s at 72°C, and 5 min at 72°C; and a final incubation step at 8°C. To determine the presence of a positive band against a known standard, the amplified PCR products (2 μl) were electrophoresed for 25 min at 100 V on 1% agarose gels stained using EcoDye™ (SolGent Co., Daejeon, Korea) and then visualized under UV illumination. The amplified PCR products were cleaned using ExoSAP-ITTM Express PCR Product Cleanup (ThermoFisher Scientific, Waltham, MA, USA) and sequenced with forward and reverse primers of both primer pairs using the Big-Dye Terminator v3.1 Cycle Sequencing Kit (Applied Biosystems, Foster City, CA, USA) and the ABI PRISM 3730xl Analyzer, (COSMO Genetech, Korea), according to the manufacturer’s protocols. Subsequently, the amplicons were sequenced using the primer sets until double-stranded coverage was achieved. ContigExpress (Vector NTI version 10.1, Invitrogen, NY, USA) was used to assemble the individual sequence reads and remove low-quality regions. The assembled sequences have been deposited in GenBank under the accession numbers PQ212526–PQ212540 (Supplementary Table S1).

### Sequence alignment and phylogenetic analysis

The SSU rDNA gene sequences of *Parvilucifera* spp. were analyzed using two isolates (accession no. PQ237103 for isolate JC041320 and PQ237102 for isolate JC042021) obtained from the same samples, as described in a previous study [29]. They were found to be 100% identical to *Parvilucifera* spp. (MG189592). For *Amoebophrya* spp., a total of 94 SSU rDNA sequences were obtained in this study from infected dinoflagellate cells (as described above), which were individually isolated across multiple time points and host species, with a focus on periods when parasitoids were most frequently observed. Among identical sequences, one representative sequence was selected for further phylogenetic analysis. SSU rDNA sequences belonging to the phylum Apicomplexa served as the outgroup. Both ends of the alignment were manually trimmed using Molecular Evolutionary Genetics Analysis 11 (MEGA11) [32], and highly divergent and poorly aligned regions were then removed using Gblocks 0.91b [33]. The final alignments of 1155 nucleotide sites were selected for further analysis. The phylogeny based on the SSU rDNA region was inferred using maximum likelihood (ML) and Bayesian inference analyses. The ML analysis was performed using IQ-TREE v1.6 [34]. The best nucleotide substitution model was determined using jModelTest 2.1.10 [35], and a transitional model with six free parameters and unequal base frequencies (GTR + I + G) was selected based on Akaike and Bayesian information criteria. Moreover, 1000 nonparametric bootstrap trees were reconstructed using the same method to assess node supports. The Bayesian analysis was performed using MrBayes v3.2.7a [36] by running four simultaneous Markov chain Monte Carlo (MCMC) chains for 2 000 000 generations. Sampling was performed every 100 generations, following a burn-in of 2000 generations.

### Niche analysis

Before performing statistical analyses, the environmental variables were standardized to values between 0 and 1 based on the minimum and maximum values of each variable. Cell densities were log transformed [ln (x + 1)] to reduce the effect of dominant species and subsequently standardized using Z-score normalization before the outlying mean index (OMI) analysis to minimize the effect of outliers and enable comparison across variables. Given the difficulty in quantifying the abundance of each parasitoid, particularly within the *Amoebophrya* complex, clade-level data were incorporated into the OMI analysis as binary presence/absence values instead of using abundance estimates. All statistical analyses were performed in R software [37] using packages freely available on the CRAN repository.

The OMI analysis is an ordination technique that characterizes the NR distribution of species in the entire community in response to environmental gradients [38]. The analysis was performed using the “niche” function of the “ade4” package [39]. The OMI analysis provides an NR bounded by the environmental space *E* defined by two environmental gradients (OMI1 and OMI2) and estimates three parameters for each taxonomic unit in the community: marginality (or OMI), tolerance (TOL), and residual tolerance (Rtol). Marginality corresponds to the Euclidean distance between the mean environmental conditions experienced by a species and the mean environmental conditions of the realized environmental space. High and low marginality values imply that a species occurs in atypical (uncommon, i.e. environmental conditions far from the mean) and typical (common, i.e. environmental conditions close to the mean) habitats, respectively. The TOL parameter refers to the niche breadth that determines the environmental range in which a species occurs. High and low TOL values correspond to the occurrence of species in a wide range (i.e. generalist species) and a limited range (i.e. specialist species) of environmental conditions, respectively [40]. The Rtol parameter determines the suitability of the environmental variables used to define the species niche [37]. The statistical significance of the OMI values was assessed using Monte Carlo permutations included in the “ade4” package (10 000 permutations).

The “envfit” function of the “vegan” package was used to fit the environmental variables into the first two OMI axes. To visualize the frequency of occurrence of each parasitoid (based on presence or absence), KDE plots were generated using the “kde” function of the “ks” package [41]. Niche overlap was subsequently assessed by comparing the NRs and calculating the D metric for each parasitoid pair using the “ecospat.niche.overlap” function of the “ecospat” package [42].

The relevance of environmental variables (e.g. temperature, salinity, and nutrients) for parasitoids was assessed by performing a marginal permutational multivariate analysis of variance (PERMANOVA) based on Euclidean distances (Supplementary Table S2) [43]. However, because of insufficient variations in the environmental data or the low abundance of *Amoebophrya*-C4 parasitoids across the samples, PERMANOVA could not be successfully performed for this group. The analysis estimated an empirical (pseudo) F-value for each term using 10 000 permutations to test the null hypothesis of no association between the parasitoids and environmental factors. This analysis was performed using the “adonis2” function of the “vegan” package [44].

## Results

### Physiochemical conditions at the monitoring site

**Figure 1:**
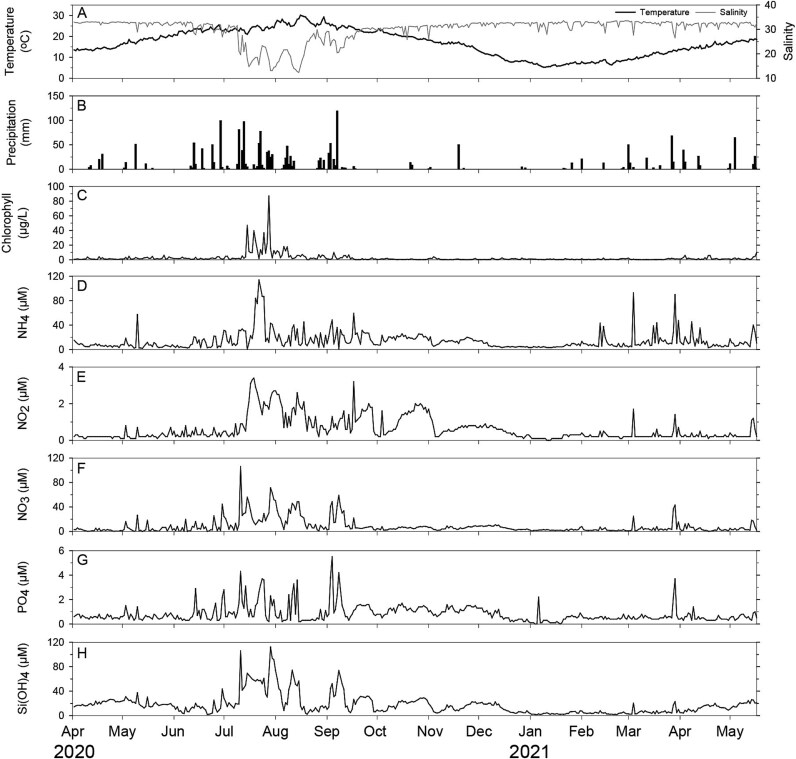
Temporal variations in environmental variables at the monitoring site in Jinhae Bay from 2 April 2020, to 17 May 2021. Daily measurements of surface water temperature and salinity (A), chlorophyll concentration (C), and concentrations of dissolved inorganic nutrients including ammonium, nitrate, nitrite, phosphate, and silicate (D–H), are presented. Daily precipitation (B) recorded in the study area is also presented.

A summary of the physicochemical conditions at the monitoring site during the sampling period from 2 April 2020, to 17 May 2021, is provided in Table 1. Surface water temperature showed a large seasonal variation, typical of a temperate coastal regime. It ranged from 5.3°C in January 2021 to 30.1°C in August 2020. As shown in Fig. 1, strong temporal fluctuations were also noted in other environmental variables, including salinity, nutrient concentrations, and precipitation. Notably, the sampling site was characterized by exceptionally heavy rainfalls during the summer season in 2020, with daily precipitation reaching up to 118.6 mm. This resulted in an atypical environment with very low salinity (12.41) (Fig. 1A and B). Moreover, a high riverine input of dissolved inorganic nutrients was noted from July to August (Fig. 1D–H and Table 1). For example, nutrient concentrations ranged from 0.26 to 113.96 μmol L^−1^ for NH_4_, 1.65 to 112.3 μmol L^−1^ for Si(OH)_4_, 0.05 to 5.51 μmol L^−1^ for PO_4_, 0.03 to 3.44 μmol L^−1^ for NO_2_, and 0.24 to 106.24 μmol L^−1^ for NO_3_.

**Table 1:** A summary of environmental variables monitored over 411 days at Jangcheon Port, Jinhae Bay, and the relationship between the environmental variables and the first two OMI axes assessed using the “envfit” function. The squared correlation coefficient (r2) values represent the proportion of variance explained by the ordination. The *P*-value (Pr) represents the significance of the correlation between the environmental variables and the ordination axes. The significance was tested using 999 permutations.

	Min	Max	Average	OMI1	OMI2	r^2^	Pr (>r)	
Temp	5.3	30.1	16.86	0.62449	0.78104	0.1295	0.001	c
Sal	12.41	33.56	30	−0.99857	−0.05343	0.1409	0.001	c
Tide	45.03	120	78.43	0.62941	−0.77707	0.0775	0.001	c
Chl	0.02	86.99	2.45	0.97616	0.21706	0.0150	0.060	d
Wind	0.56	7.13	1.99	−0.97388	−0.22707	0.0180	0.034	a
Pre	0	118.6	0.20	0.97938	−0.20203	0.0102	0.140	e
Sta	−7.8	8.73	0.06	−0.53566	0.84443	0.0039	0.459	e
NO_2_	0.03	3.44	0.62	0.99569	0.09273	0.1617	0.001	c
NO_3_	0.24	106.24	8.5	0.99660	0.08239	0.0761	0.001	c
NH_4_	0.26	113.96	13.92	0.99051	0.13742	0.0555	0.001	c
SiOH_4_	1.65	112.3	17.94	0.68221	0.73116	0.0262	0.006	b
PO_4_	0.05	5.51	0.83	0.99979	0.02056	0.0775	0.001	c

Significance codes:

a *P* < .05.

b *P* < .01.

c *P* < .001.

d *P* < .1.

e *P* < 1.

*Abbreviations:* Temperature (Temp, °C), salinity (Sal), tidal coefficient (Tide), chlorophyll fluorescence (Chl, μg L ^−1^), wind speed (Wind, m s ^−1^), precipitation (Pre, mm), and stability ratio (Sta).

### Temporal dynamics of dinoflagellate hosts and their two parasitoids

**Figure 2:**
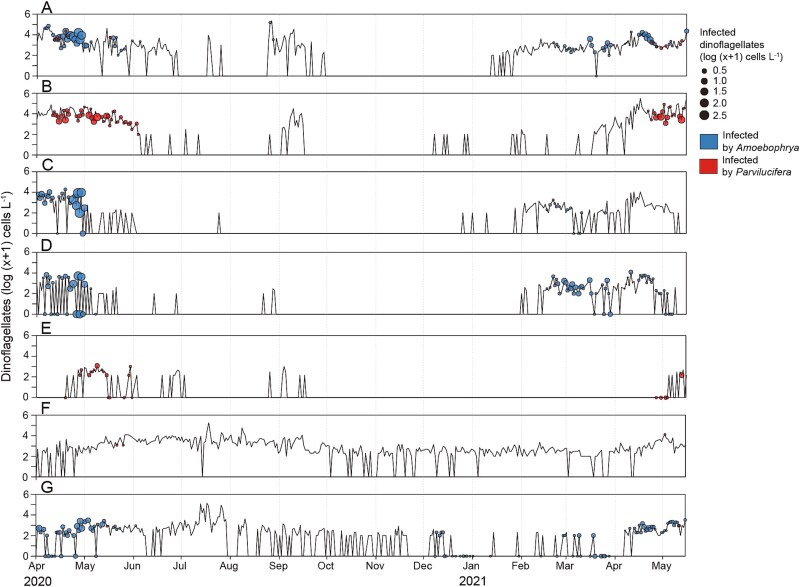
Temporal dynamics of dinoflagellate hosts and their cells infected by the two parasitoids over a 411-day sampling period. Solid black lines indicate host abundance (log-transformed cell density), while colored circles represent infected host cells (blue represents *Amoebophyra* infections and red represents *Parvilucifera* infections). (A) *Alexandrium* spp., (B) *Akashiwo sanguinea*, (C) *H. triquetra*, (D) Scrippsielloids, (E) *P. steinii*, (F) *Protoperidinium* spp., and (G) *Dinophysis acuminata*.

The temporal dynamics of the hosts and their two parasitoids exhibited pronounced seasonal fluctuations during the sampling period (Fig. 2). Two distinct temporal occurrence patterns of the hosts were identified: [1] taxa observed throughout the year and [2] taxa detected only during specific seasons. Although *Protoperidinium* spp. and *Dinophysis acuminata* were observed throughout the year (Fig. 2F and G), they were predominantly infected by parasitoids from April to May. However, a rare infection of *D. acuminata* by *Amoebophrya* spp. was detected in December. In contrast, hosts that appeared only during specific seasons were mostly present in high abundance from March to May (Fig. 2A–E). Notably, *Alexandrium* spp. (Fig. 2A) and *Akashiwo sanguinea* (Fig. 2B) exhibited similar temporal occurrence patterns. Similarly, Scrippsielloids spp. and *Heterocapsa triquetra* exhibited comparable patterns, characterized by elevated abundance between February and May (Fig. 2C and D). Although the abundance of *Pyrophacus steinii* was lower than that of other taxa, it began to emerge in late April (Fig. 2E). Most infections by the two parasitoids were detected from March and May, with occasional infections by *Amoebophrya* spp. observed in September and December.

**Figure 3:**
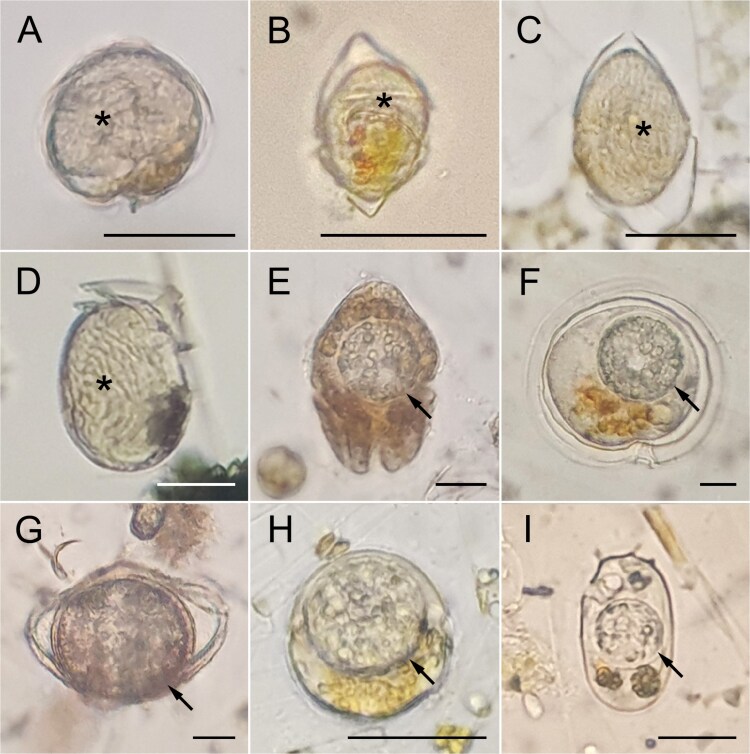
Dinoflagellate cells infected by the parasitoids *Amoebophrya* spp. (A–D) and *Parvilucifera* spp. (E–I). (A) *Alexandrium* spp., (B) *H. triquetra*, (C) Scrippsielloids, (D) *Dinophysis acuminata*, (E) *Akashiwo sanguinea*, (F) *P. steinii*, (G) *Protoperidinium* spp., (H) *Alexandrium* spp., and (I) *D. acuminata*. All arrows indicate *Parvilucifera* spp., and all asterisks indicate *Amoebophrya* spp.

Throughout the 411-day monitoring period, the parasitoids were consistently found to infect dinoflagellate hosts; however, the infection ratio (%) remained relatively low. Nevertheless, temporal fluctuations were apparent, with distinct peaks in infection ratio occurring during specific periods (Supplementary Fig. S2). Parasitoid infections were restricted to dinoflagellates. However, only seven dinoflagellate species were found to be infected during the monitoring period (Fig. 3). Scrippsielloids, *H. triquetra*, *D. acuminata,* and *Alexandrium* spp. were infected by *Amoebophrya* spp., while *A. sanguinea*, *D. acuminata*, *S. acuminata*, *P. steinii*, and *Protoperidinium* spp. were infected by *Parvilucifera* spp. Of these, only *Alexandrium* spp. and *D. acuminata* were infected by both parasitoids (Fig. 3A, D, H, and I).

### Phylogeny of parasitoids

**Figure 4:**
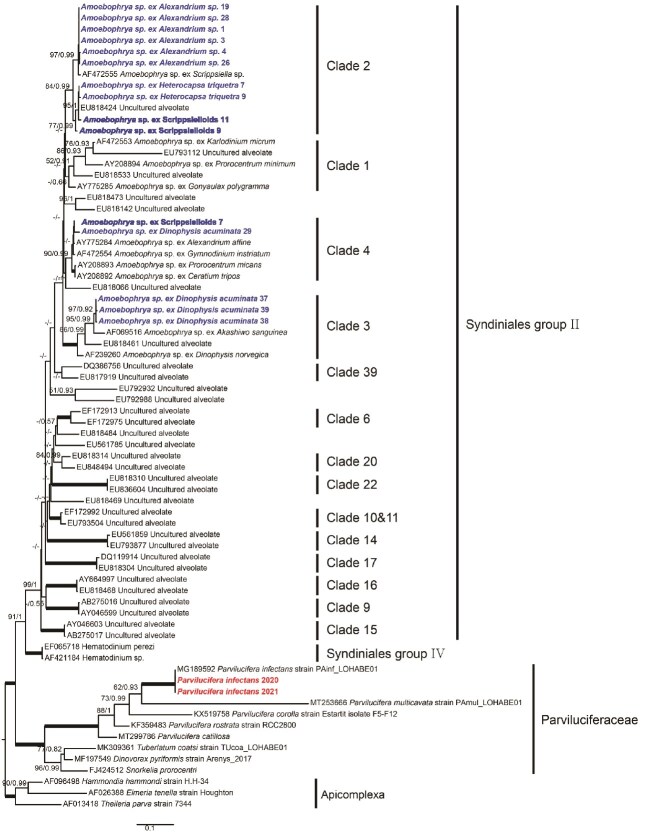
ML phylogenetic tree inferred from SSU rRNA gene sequences. The sequences for *Amoebophrya* and *Parvilucifera* spp. are indicated in blue and red, respectively. The phylogenetic analysis was conducted using a representative sequence selected from those with matching nucleotide sequences (Supplementary Table S1).

As shown in Fig. 4, the phylogenetic analysis revealed that all *Amoebophrya* sequences from the infected dinoflagellate host cells individually isolated from environmental samples belonged to only three clades (*Amoebophrya*-C2, -C3, and -C4) in the family Amoebophyraceae (Syndiniales group II), according to the classification proposed by Guillou *et al.* (2008) [45]. Of these, 51 *Amoebophrya* sequences were assigned to clade 2, 40 to clade 3, and three to clade 4, each supported by bootstrap values of >70% and posterior probabilities of >0.9. Although some clades were not fully supported by bootstrap analyses, the tree topologies remained identical across both ML and Bayesian inference analyses. The obtained *Parvilucifera* sequences were compared with previously reported sequences of *P. infectans* strain PAinf_LOHABE01 (MG189592) belonging to the family Parviluciferaceae within the phylum Perkinsozoa; these sequences were found to be identical.

*Amoebophrya* sequences were obtained from four host dinoflagellates: Scrippsielloids, *Alexandrium* spp., *H. triquetra*, and *D. acuminata*. Although these sequences were obtained from different hosts, they did not vary significantly among the hosts. The sequences were grouped into three clades (*Amoebophrya*-C2, -C3, and -C4). Some clades were detected across multiple host species, indicating that clade affiliation was not strictly determined by host identity. For example, *Amoebophrya*-C2 included sequences from *Alexandrium* spp., *H. triquetra*, and Scrippsielloids, while *Amoebophyra*-C3 primarily included sequences from *D. acuminata.*

### Patterns of host use and niche differentiation

**Figure 5:**
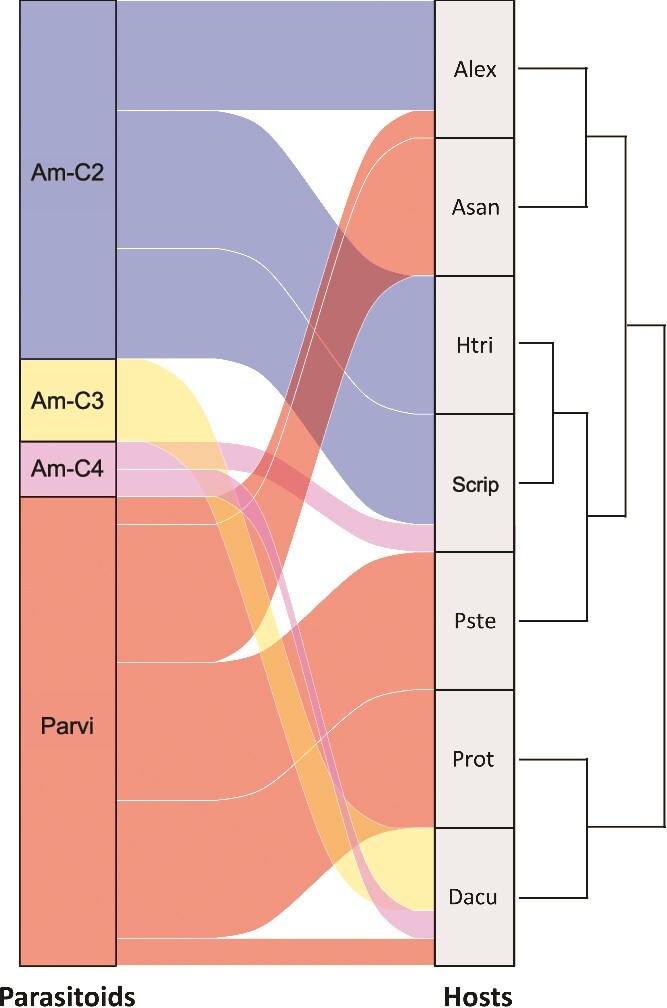
Interactions between parasitoids (left) and host dinoflagellates (right) during the study period. The width of flow from a parasitoid to a host dinoflagellate represents the extent of infection of the host by the respective parasitoid. The host species on the right are clustered based on their similar occurrence patterns during the monitoring period.

To further explore the relationship between parasitoids and their dinoflagellate hosts during the monitoring period, infection patterns were visualized (Fig. 5). The results revealed that hosts with similar seasonal dynamics were often infected by different parasitoid clades (Fig. 5). For example, although *Alexandrium* spp. and *A. sanguinea* exhibited overlapping seasonal occurrences, they were predominantly infected by different parasitoids (*Amoebophyra*-C2 and *Parvilucifera* spp., respectively). Moreover, although *Protoperidinium* spp. and *D. acuminata* exhibited overlapping seasonal occurrences, they were infected by different parasitoids (*Parvilucifera* and *Amoebophrya* spp., respectively). However, in contrast to the divergent infection patterns described above, also in some cases, hosts with similar seasonal dynamics were infected by the same parasitoid. For example, both *H. triquetra* and *S. acuminata* exhibited overlapping seasonal occurrences and were infected by *Amoebophrya*-C2.

### Niche analysis of hosts and parasitoids

**Figure 6:**
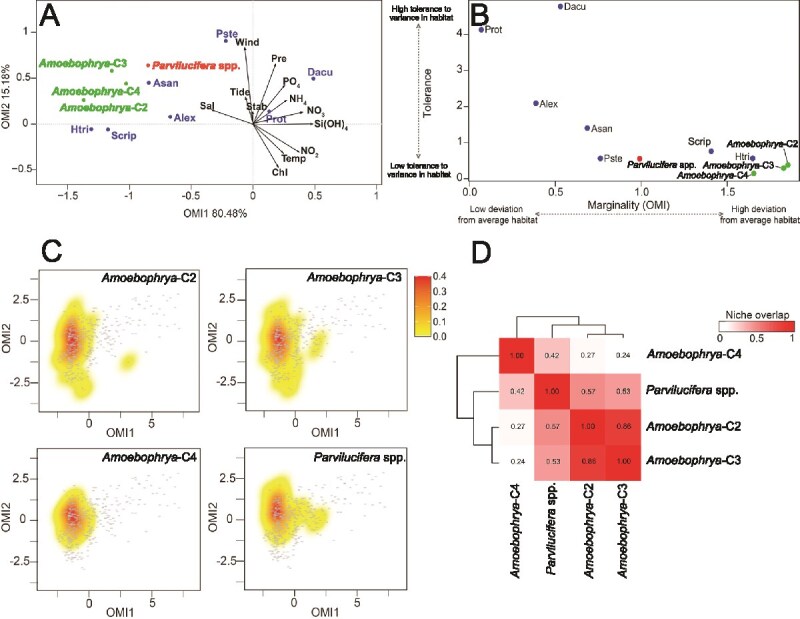
OMI analysis of host dinoflagellates and parasitoids. (A) Representation of the significant NR positions of species on the first two factorial axes with the canonical weights of environmental variables [Sal, salinity; Tide, tidal coefficient; Wind, wind speed; Pre, precipitation; Stab, stability ratio; PO_4_, phosphate concentration; NH_4_, ammonium concentration; NO_3_, nitrate concentration; NO_2_, nitrite concentration; Si(OH)_4_, silicate concentration; Temp, seawater temperature; and Chl, chlorophyll fluorescence]. The niche positions of the host dinoflagellates are indicated in blue. The parasitoids *Amoebophrya*-C2 (C2), *Amoebophrya*-C3 (C3), and *Amoebophrya*-C4 (C4) are represented in green, while *Parvilucifera* spp. (Pinf) is represented in red. The host dinoflagellate species are identified by their codes in Supplementary Table S3. (B) Total marginality (i.e. ecological niche deviation from the average environmental conditions) versus total TOL (i.e. capacity to adjust to any variance in habitat composition) of host dinoflagellates and parasitoids. (C) Distribution of the KDE of the parasitoids in the OMI multivariate space. The color gradient from yellow to red represents low to high density levels, respectively, while the gray dots represent the environmental samples. (D) A heatmap illustrating the similarities between parasitoids based on the pairwise *D* metric (i.e. niche overlap), which was calculated based on the KDE presented in C.

Preliminary analyses were conducted using all sample data (*n* = 411). To avoid misinterpretation due to missing environmental variable data, several samples were excluded from the posterior analysis (*n* = 384). The first and second axes of the OMI ordination accounted for 95.66% of the total projected inertia (OMI1: 80.48% and OMI2: 15.18%) (Fig. 6A). On the OMI1 axis, the positive side was positively correlated with concentrations of dissolved inorganic nutrients [NO_3_, NO_2_, and Si(OH)_4_] and temperature and negatively correlated with salinity. The OMI2 axis was primarily correlated with wind speed. The *envfit* test revealed that the variable seawater temperature, salinity, and NO_2_ concentration accounted for most of the total explained variability (R^2^ = 0.1295, 0.1409, and 0.1617, respectively; *P* < .01; Table 1).

The OMI analysis indicated that the niches of the hosts and parasitoids were characterized by species-specific environmental preferences (Fig. 6A). The niche positions of most host dinoflagellates and their parasitoids were predominantly influenced by salinity and therefore shifted leftward on the OMI plot, with the exceptions of three hosts: *D. acuminata*, *Protoperidinium* spp., and *P. steinii*. In other words, as a result of high nutrient concentrations and low salinity caused by frequent rainfall, most host dinoflagellates and their parasitoids were positioned in the upper left quadrant of the OMI plot. Moreover, host dinoflagellates and their parasitoids were negatively correlated with temperature, Si(OH)_4_ concentration, and NO_2_ concentration (Fig. 6A). Thus, summer-like conditions with frequent rainfall, low salinity, and high nutrient concentrations seem to be unfavorable for host dinoflagellates and their parasitoids.

Marginality (i.e. OMI) is determined by the deviation from a theoretical species that is uniformly distributed across all available habitat conditions (OMI = 0). This measure is inversely correlated with the TOL index. All host dinoflagellates and parasitoids exhibited significant OMI values (*P* < .05), except for *P. steinii* (*P* > .05) (Fig. 6B and Supplementary Table S3). The most uniformly distributed species were *Protoperidinium* spp. and *D. acuminata*, exhibiting OMI values of 0.06 and 0.53 and TOL values of 4.12 and 4.75, respectively. On the other hand, the three *Amoebophrya* clades (i.e. *Amoebophrya*-C2, -C3, and -C4) were the most specialized species, followed by *H. triquetra* and *S. acuminata* (Supplementary Table S3). In the OMI multivariate space, *Protoperidinium* spp. and *D. acuminata* occupied large portions of the realized environmental space (Supplementary Fig. S3A).

The parasitoids were considered ecologically restricted; they significantly diverged from those of theoretically ubiquitous species. Moreover, they exhibited a narrower niche breadth than their host dinoflagellates (Fig. 6B and Supplementary Fig. S3B). Among the *Amoebophrya* parasitoids, *Amoebophrya*-C4 exhibited the narrowest NR breadth (TOL = 0.11), while *Amoebophrya*-C2 exhibited the widest NR breadth (TOL = 0.35) (Supplementary Fig. S3B and Table S3). The NR breadth of *Parvilucifera* spp. (OMI = 0.99 and TOL = 0.54) was wider than that of *Amoebophrya* spp. The positions of these niches within the environmental space revealed partial separation among the four parasitoids despite the concentration of the mean habitat condition center points.

The kernel density estimates (KDE) for the four parasitoids plotted on the first two OMI axes (Fig. 6C) revealed patterns related to the presence and absence of the parasitoids in relation to environmental factors. The parasitoids showed similar NRs throughout the sampling period. In particular, the density of *Amoebophrya*-C2 and -C3 was high near the center of both axes. Moreover, their density distribution was remarkably similar. This suggests that they occupy a similar niche space. However, *Amoebophrya*-C4 exhibited a more restricted pattern, appearing slightly higher on the OMI2 axis than *Amoebophrya*-C2 and -C3. This suggests the presence of different but overlapping environmental distributions. In contrast, *Parvilucifera* spp. exhibited a similar pattern to *Amoebophrya*-C2 and -C3; however, their density distribution extended over a broader range along the OMI1 axis (Fig. 6C). The heatmap revealed the degree of niche overlap among the four parasitoids (Fig. 6D); the values ranged from 0 to 1, with higher values indicating greater overlap in the niche space. The dendrogram in the heatmap indicates the clustering of parasitoids based on their niche overlap. *Amoebophrya*-C2 and -C3 exhibited the highest niche overlap (0.86), suggesting that they share a similar habitat. *Parvilucifera* and *Amoebophrya* spp. exhibited moderate niche overlap (0.42–0.57), indicating some shared environmental conditions along with distinct occurrence patterns. *Amoebophrya*-C4 showed lesser niche overlap with the other parasitoids. The KDE plots of parasitoid occurrence across environmental variables support the niche distributions and overlap patterns observed among the parasitoids (Supplementary Fig. S4). All parasitoids were associated with low nutrient concentrations; however, an exception was observed in the Si(OH)_4_ plot (Supplementary Fig. S4L), where the species exhibited different patterns. *Amoebophrya*-C2 and -C3 were frequently detected at concentrations below 10 μmol L^−1^, while *Amoebophrya*-C4 and *Parvilucifera* spp. were only detected at higher concentrations. Moreover, *Parvilucifera* spp. were more frequently detected at temperatures higher than 12°C, while *Amoebophrya*-C2 and -C3 were detected at a broader temperature range (10°C–16°C) (Supplementary Fig. S4A).

## Discussion

The major findings of the present study are as follows: [1] *Amoebophrya* spp. belonging to at least three genetic clades and *Parvilucifera* spp. coexisted at the monitoring site. [2] Although all parasitoids examined in this study exhibited low TOL (i.e. narrow niche breadth), they exhibited different niche positions, ranging from high marginality (*Amoebophrya*-C2, -C3, and -C4) to moderate marginality (*Parvilucifera* spp.). [3] *Amoebophrya*-C2 and -C3 exhibited high niche overlap, while *Amoebophrya*-C4 exhibited low niche overlap with the other parasitoids. [4] Host niche partitioning was observed among the parasitoids infecting dinoflagellates. These findings provide insights into the niche position and breadth of the parasitoids that infect dinoflagellates and facilitate further assessment of their relationships through a niche overlap metric.

The OMI analysis revealed distinct niche properties of *Amoebophrya* and *Parvilucifera* parasitoids, which use dinoflagellates as hosts. Interestingly, niche position differed between these parasitoids. In previous study applying OMI analyses, species with higher occurrence frequency were found to exhibit lower marginality and higher TOL [15]. This general trend provides an ecological framework for interpreting the niche properties observed in the present study. Although all parasitoids examined in the present study exhibited low TOL (i.e. narrow niche breadth), they exhibited different niche positions. *Amoebophrya* spp. (i.e. *Amoebophrya*-C2, -C3, and -C4) exhibited high marginality, suggesting that they occur in less common environmental conditions at the study site. This combination of high marginality and low TOL suggests that these parasitoids are confined to a narrow range of environmental conditions that substantially differ from the habitat mean, reflecting ecological specialization rather than broad adaptability. In contrast, *Parvilucifera* spp. exhibited moderate marginality, implying that they occur in relatively typical environmental conditions compared to *Amoebophrya* spp. Thus, the observed difference in marginality between these parasitoids suggests that *Parvilucifera* infections could be more frequent than *Amoebophrya* infections at the study site. While the OMI analysis primarily reflects niche properties based on environmental factors, the observed difference in marginality may be partially influenced by biological factors, such as host specificity. Parasitoids with strong host specificity may be heavily influenced by the environmental conditions of the habitat where their specific hosts exist. Consequently, *Amoebophrya* spp., which are highly specialized to certain hosts (Fig. 5), may be restricted to environments where these hosts exist. On the other hand, *Parvilucifera* spp., which have a broader host range, can occupy a wider range of environmental conditions, resulting in relatively lower marginality. The high marginality values observed for *Amoebophrya* spp. may thus be interpreted as being related to the degree of their host specificity. Together, these findings support the interpretation that NR positions of parasitoids are strongly influenced by their host range. A narrow host range, as observed in certain *Amoebophrya* clades, may limit ecological opportunities and lead to higher marginality. On the other hand, a broader host range, as observed in *Parvilucifera* spp., may allow greater ecological flexibility and enable access to more common environmental conditions. However, this marginality may partly reflect time-lagged responses to host availability that are not captured by the static nature of the OMI analysis.

Parasitoids and hosts are in a physically close relationship, regardless of whether the parasitoids are endoparasitoids or ectoparasitoids. The parasitoids used for analyzing niche properties in the present study were derived from infected hosts and not zoospores at the free-living stage. Hence, the parasitoids would be expected to have similar niche properties as their hosts in the field. However, this was not observed in the parasitoids investigated in the present study. For example, *Amoebophrya*-C3, which was retrieved from only *D. acuminata* cells, exhibited high marginality and low TOL, while its host (*D. acuminata*) exhibited relatively low marginality and high TOL. Furthermore, the *D* metric (i.e. niche overlap) between them was calculated to be very low (0.36), supporting differences in their niche properties. In the case of *Parvilucifera* spp., which infected five dinoflagellates, the hosts varied from generalists with high TOL (i.e. *D. acuminata*) to specialists with low TOL (i.e. *P. steinii*). These results could be explained by at least two factors. First, abiotic environmental conditions encountered during different life cycle stages, such as the zoospore stage, may be more important for determining the ecological niche of a parasitoid than the resources provided by the living body of its host. PERMANOVA identified salinity, tidal effect, and nutrient concentrations as significant abiotic factors influencing the distribution of parasitoids, although these factors accounted for only a small portion (~15%) of the observed variance (Supplementary Table S2). While *Parvilucifera* spp. may be influenced by factors beyond the considered environmental variables (Rtol = 5.06; Supplementary Table S3), the strong association between their ecological distribution and the examined environmental factors suggests their significant impact. Thus, even if hosts are consistently available, the parasitoids will not thrive unless the environmental conditions are favorable. Only a few studies have assessed the impact of environmental variables, such as nutrient concentrations and water temperature, on *Amoebophrya* and *Parvilucifera* spp. [46, 47], and research in this field is lacking. Nonetheless, the intrinsic biological properties of the parasitoids cannot be ignored.

Second, the ecological niche of parasitoids may be influenced by predation pressure exerted by predators. Parasitoids are vulnerable to predation; therefore, their ecological niche can be narrower than that of their hosts, even when hosts are present. A few studies have documented that *Amoebophrya* and *Parvilucifera* spp. can be consumed by predators at the free-living zoospore stage [48, 49]. For example, under controlled culture conditions, the choreotrich ciliate *Strobilidium* sp. was found to rapidly ingest and digest infective dinospores of *Amoebophrya* sp. ex *A. sanguinea* [48]. Laboratory experiments also revealed that grazing by *Strobilidium* sp. could reduce the infection rate of *A. sanguinea* by 70%–80% relative to controls [48]. Thus, in environments with high predation pressure, parasitoids may be more frequently found in safer, specific ecological niches where exposure to predators is minimized. Such predation pressure could be a contributing factor to the specialization of parasitoids to certain hosts or their occupation of specific ecological niches.

KDE plotted on the first two OMI axes indicated that *Amoebophrya* and *Parvilucifera* spp. coexisting at the monitoring site shared similar NRs. These parasitoids would be expected to compete for the same resource and not coexist for a long period, according to Gause’s law (also known as the principle of competitive exclusion) [50]. However, to reduce and/or avoid competition, competing species can differentiate their niches in many ways, such as spatially (e.g. by consuming different foods or using different areas within the environment), temporally (e.g. by using the same resource at different times), or morphologically. Such niche partitioning has also been noted in phytoplankton [13, 14, 18, 19, 51]. For example, Baldrich *et al.* (2021) [18] recently reported spatial (i.e. vertical) niche partitioning of two coexisting *Dinophysis* spp. (*D. acuta* and *D. acuminata*) in the water column of a stratified fjord in Chile. Moreover, Min and Kim (2023) [19] reported seasonal niche changes in three *Alexandrium* spp. in the Korea Strait, with *Alexandrium catenella* exhibiting the highest abundance in spring, *A. pacificum* in summer, and *A. affine* in autumn. In the present study, the parasitoids seemed to infect different dinoflagellate hosts to coexist and/or reduce competition through host niche partitioning. The heatmap based on the niche overlap analysis revealed a considerable variation in niche overlap (0.24–0.86) among these parasitoids. For instance, the highest niche overlap (0.86) was noted between *Amoebophrya*-C2 and -C3, suggesting the existence of strong competition between them for the same resource (i.e. dinoflagellate host). In this case, *Amoebophrya*-C2 was found to infect the dinoflagellates *Alexandrium* spp., *H. triquetra*, and Scrippsielloids, while *Amoebophrya*-C3 was found to infect only *D. acuminata* (Fig. 5). In comparison, *Amoebophrya*-C4, which was detected in the hosts *S. acuminata* and *D. acuminata*, exhibited very low niche overlap with other *Amoebophrya* spp. (C2 and C3) (0.24–0.27) and *Parvilucifera* spp. (0.42). *Parvilucifera* spp. exhibited moderate niche overlap (0.42–0.57) with all *Amoebophrya* spp. examined in the present study. They used *A. sanguinea*, *P. steinii*, and *Protoperidinium* spp. as their main hosts, and they occasionally used *Alexandrium* spp. and *D. acuminata* as hosts. These patterns highlight how host niche partitioning among these parasitoids may facilitate their coexistence by reducing direct competition for the same host resources.

Our results demonstrate that the coexistence of *Amoebophrya* and *Parvilucifera* spp. is facilitated by niche partitioning through the use of different dinoflagellate hosts. These results highlight how ecological differences and adaptive strategies, such as infecting different hosts, contribute to niche differentiation and coexistence. Future research should focus on the role of environmental and biological factors, such as predation pressure, in shaping niche dynamics and interactions among marine parasitoids.

## Supplementary Material

Sup_Fig_1_ycaf126

Sup_Fig_2_ycaf126

revised_Sup_Fig_3_ycaf126

revised_Sup_Fig_4_ycaf126

revised_Supplementary_Table_S1_ycaf126

Supplementary_Table_S2_ycaf126

revised_Supplementary_Table_S3_ycaf126

## Data Availability

The nucleotide sequences generated during this study have been deposited in the GenBank repository and are accessible through the following accession numbers: PQ212526–PQ212540.
