# Supplementary figures and images for: Host niche partitioning and coexistence in Amoebophrya and Parvilucifera parasitoids infecting dinoflagellates
Source: ISME Commun. 2025 Jul 29;5(1):ycaf126. doi: 10.1093/ismeco/ycaf126 (PMC12343117; doi:10.1093/ismeco/ycaf126)

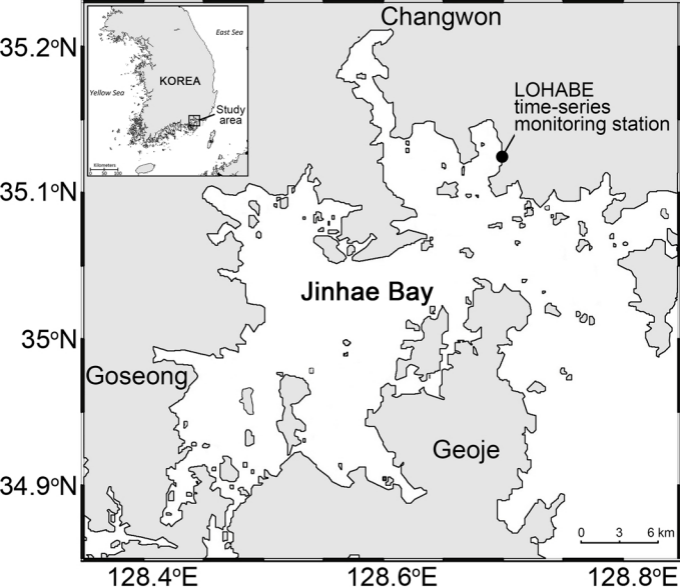

Supplement: Sup_Fig_1_ycaf126 [file sup_fig_1_ycaf126.pdf]

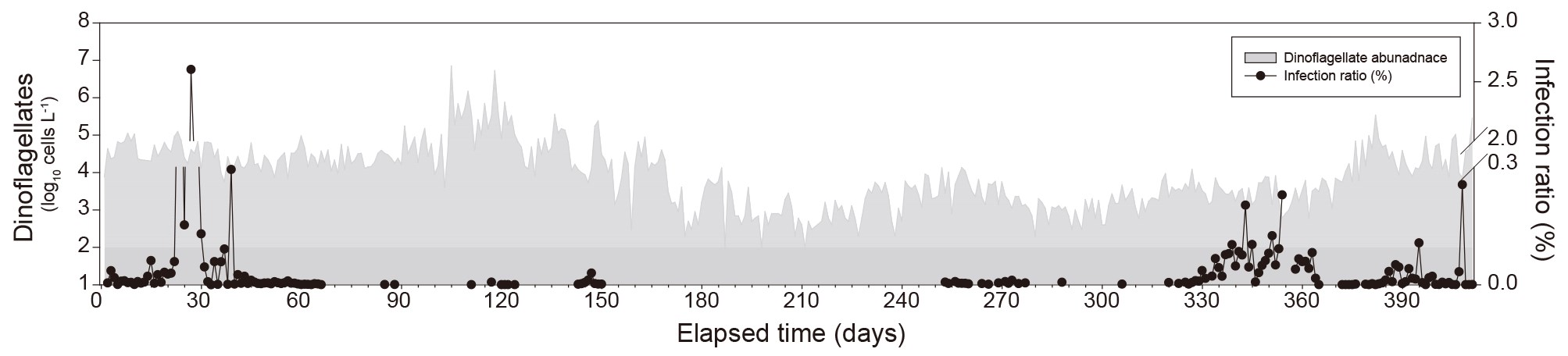

Supplement: Sup_Fig_2_ycaf126 [file sup_fig_2_ycaf126.jpeg]

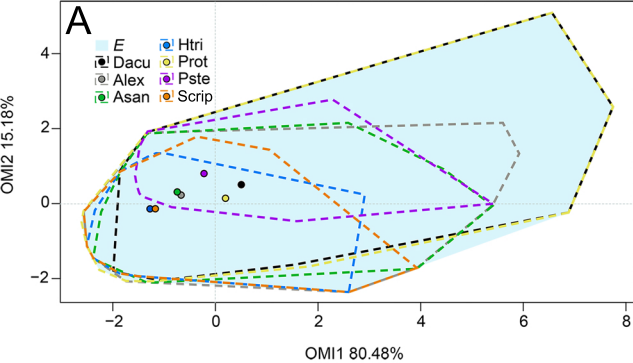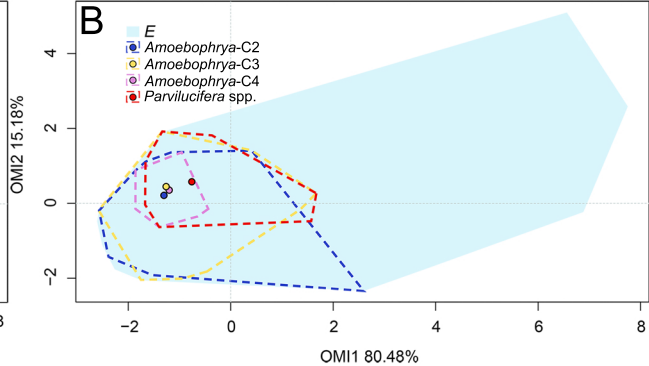

Supplement: revised_Sup_Fig_3_ycaf126 [file revised_sup_fig_3_ycaf126.pdf]

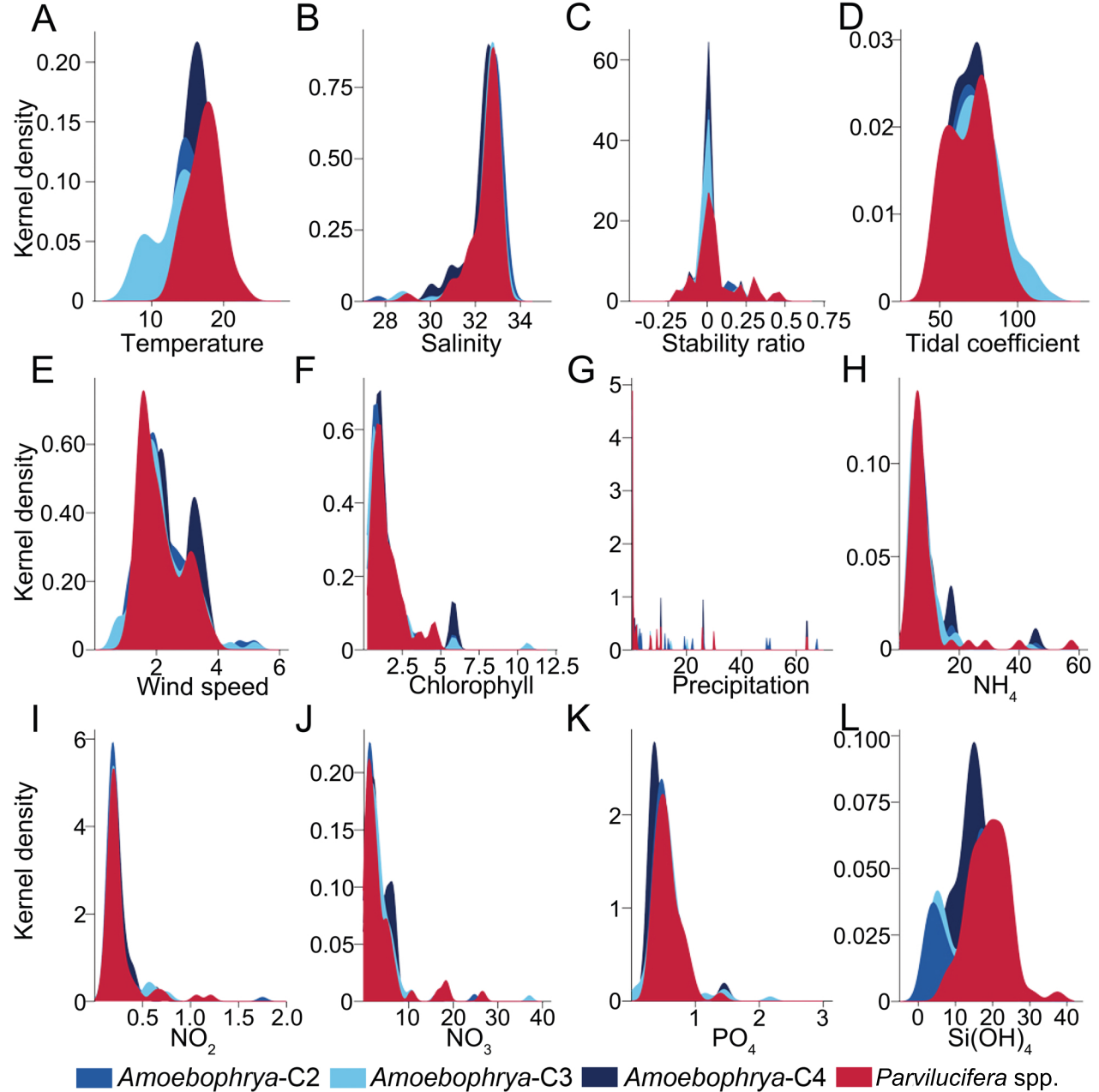

Supplement: revised_Sup_Fig_4_ycaf126 [file revised_sup_fig_4_ycaf126.pdf]
